# Expression of DDX11 and DNM1L at the 12p11 Locus Modulates Systemic Lupus Erythematosus Susceptibility

**DOI:** 10.3390/ijms22147624

**Published:** 2021-07-16

**Authors:** Mohammad Saeed, Alejandro Ibáñez-Costa, Alejandra María Patiño-Trives, Laura Muñoz-Barrera, Eduardo Collantes Estévez, María Ángeles Aguirre, Chary López-Pedrera

**Affiliations:** 1 ImmunoCure, Karachi 75500, Pakistan; msaeed@immunocure.pk; 2 Rheumatology Service, Maimonides Institute for Biomedical Research of Cordoba (IMIBIC), Reina Sofia Hospital, University of Cordoba, 14004 Cordoba, Spain; b12ibcoa@uco.es (A.I.-C.); alejandramaria.patino@gmail.com (A.M.P.-T.); b62mubal@uco.es (L.M.-B.); educollantes@yahoo.es (E.C.E.); maaguirrezamorano@yahoo.es (M.Á.A.)

**Keywords:** lupus, DDX11, DNM1L, KRAS, OASIS

## Abstract

Objectives: This study employed genetic and functional analyses using OASIS meta-analysis of multiple existing GWAS and gene-expression datasets to identify novel SLE genes. Methods: Four hundred and ten genes were mapped using SNIPPER to 30 SLE GWAS loci and investigated for expression in three SLE GEO-datasets and the Cordoba GSE50395-dataset. Blood eQTL for significant SNPs in SLE loci and STRING for functional pathways of differentially expressed genes were used. Confirmatory qPCR on SLE monocytes was performed. The entire 12p11 locus was investigated for genetic association using two additional GWAS. Expression of 150 genes at this locus was assessed. Based on this significance, qPCRs for DNM1L and KRAS were performed. Results: Fifty genes were differentially expressed in at least two SLE GEO-datasets, with all probes directionally aligned. DDX11, an RNA helicase involved in genome stability, was downregulated in both GEO and Cordoba datasets. The most significant SNP, rs3741869 in OASIS locus 12p11.21, containing DDX11, was a cis-eQTL regulating DDX11 expression. DDX11 was found repressed. The entire 12p11 locus showed three association peaks. Gene expression in GEO datasets identified DNM1L and KRAS, besides DDX11. Confirmatory qPCR validated DNM1L as an SLE susceptibility gene. DDX11, DNM1L and KRAS interact with each other and multiple known SLE genes including STAT1/STAT4 and major components of IFN-dependent gene expression, and are responsible for signal transduction of cytokines, hormones, and growth-factors, deregulation of which is involved in SLE-development. Conclusion: A genomic convergence approach with OASIS analysis of multiple GWAS and expression datasets identified DDX11 and DNM1L as novel SLE-genes, the expression of which is altered in monocytes from SLE patients. This study lays the foundation for understanding the pathogenic involvement of DDX11 and DNM1L in SLE by identifying them using a systems-biology approach, while the 12p11 locus harboring these genes was previously missed by four independent GWAS.

## 1. Introduction

Systemic lupus erythematosus (SLE) is a complex disorder manifesting as a syndrome [1]. In SLE, identification of susceptibility genes is of great relevance for understanding specific pathobiological mechanisms and expanding the number of molecular targets for clinical testing and drug discovery. Despite the identification of a large number of genes in multiple SLE genome-wide association studies (GWAS) and candidate gene studies, together they explain only 15 to 30% of SLE heritability [2,3,4]. It is possible that several loci of modest significance remain to be discovered due to the complex, syndromic nature of SLE, along with other GWAS limitations. Multiple strategies have been applied to tease out such modest genetic effects including GWAS meta-analyses, inclusion of different populations, gene-based testing and biological pathway analysis [2,4].

A complex disease such as SLE could be thought of as a mixture of multiple resembling phenotypes, each a result of a separate mutation/susceptibility variant, pooled in GWAS cohorts [2]. Finding a particular gene, then, depends on the enrichment of a causal variant carrying haplotype in the study sample. Genotyping a multitude of single-nucleotide polymorphisms (SNPs) in GWAS leads to multiple testing and proportional signal problems, preventing modest associations from being distinguished from random noise. GWAS have majorly contributed to genetic discovery of complex disorders; however, genotyping an enormous number of variants leads to a high rate of false positivity. This is dealt with by multiple testing corrections, which directly result in a high false negativity rate leading to missing heritability, a major cause of disappointment with GWAS. The other approach to deal with the inherent false positivity has been association binning methods such as gene-based testing. Such tests generally work by assigning the most significant, or a weighted *p* value to a gene. Binning methods, by decreasing the number of tests per study, potentially increase power and reduce the multiple-testing burden. Caveats with such approaches include skewing of the association signal due to weighting procedures and to the large extent of LD in historically more recent mutations. OASIS, based on linkage disequilibrium (LD) clustering (Available online: https://ldlink.nci.nih.gov/?tab=ldmatrix (accessed on 10 May 2021)), can be used to mine existing GWAS datasets for new genes [2,5]. OASIS provides an alternative to increasing sample size for GWAS by composite analysis, unifying two aspects of the LD phenomenon: strength of association and number of surrounding significant SNPs. A genomic convergence approach [6] mapping genetic association signals on expression data, and other biological studies, can then be used for verification of particular genes. The concept is that multiple datasets with results pointing in the same direction is evidence of a true scientific finding [6]. In genomic studies, the most frequent datasets that have been converged are genetic association studies highlighting a candidate gene and the expression of that gene in a biologically relevant tissue.

We mapped 410 genes to 30 SLE loci identified using OASIS analysis of two dbGAP GWAS datasets (6077 subjects; 0.75 million SNPs) [2]. Using a genomic convergence approach, these genes were investigated for expression in three SLE GEO datasets and a fourth from Cordoba, Spain. This methodology identified DDX11, located at the 12p11 locus, as an SLE susceptibility gene. The most significant SNP in GWAS is an eQTL modulating expression of DDX11. DDX11 is an RNA helicase involved in genome stability and is repressed in SLE. Since the 12p11 locus has previously been linked to familial SLE as well, we performed a comprehensive screen of the 12p11 locus (~50K SNPs) in two additional GWAS. This study confirmed DDX11 and further identified DNM1L and its eQTLs to be associated with SLE. DNM1L, involved in mitophagy, is upregulated in SLE. The expression of both DDX11 and DNM1L was confirmed to be significantly altered in SLE by quantitative PCR on SLE monocytes.

Convergence of results from several datasets of multiple data types, such as genetic association and expression, provides robust evidence of pathobiological involvement of genes. This study, by utilizing genomic convergence of the 12p11 locus with four European GWAS and four gene expression datasets, followed by eQTL and protein network analysis, identified DDX11 and DNM1L as novel SLE susceptibility genes.

## 2. Results

### 2.1. Global Genomic Convergence

SNIPPER located 410 genes (Table S1) in 30 SLE loci identified by OASIS 2, and these were tested in three GEO expression datasets. Over 1000 expression probe sets in GEO datasets GSE30153, GSE13887 and >500 probe sets in GEO dataset GSE10325 were identified for the reference gene list. Significant *t*-tests narrowed the search to 103 probes in dataset 4193 (GSE30153), 141 probes in dataset 4719 (GSE13887) and 66 probes in dataset 4185 (GSE10325). An additional 215 probes were found to be significant in GSE10325: T-cells, B-cells and Monocytes datasets. Hence, a total of 525 statistically significant probe sets in six GEO datasets were finalized. This composite probe list was matched against the SNIPPER reference gene list of 410 genes to remove those that the GEO query included, but were not found in the OASIS SLE loci. Hence, 187 unique genes in the six GEO datasets were identified that had significant changes in expression and were located in the 30 SLE loci (data not shown).

Of the 187 candidate genes, 55 genes were found to be differentially expressed in at least two GEO datasets with all probes directionally aligned (Table S2). Ten genes crossed the false discovery rate (FDR) and seven crossed the Bonferroni correction (Table S2). DDX11, located in OASIS locus 19 (12p11.21), was downregulated but did not cross the FDR for expression. In the Cordoba expression dataset, GSE50395, 518 genes were found to have differential expression. Of these, 10 matched with the reference list of 410 genes (Table S2). BARHL1 had the most significant expression change (*p* = 8.9 × 10^−5^) and DDX11 had the second highest expression change (*p* = 8 × 10^−3^).

### 2.2. eQTL Analysis

In six of 10 genes identified in the Cordoba dataset (GSE50395), eQTLs were detected. The most significant SNP rs3741869 (db1 GWAS *p* = 3.2 × 10^−5^) in OASIS locus 19 (12p11.21), containing the gene DDX11, was found to be a cis-eQTL regulating the expression of DDX11 (*p* = 8.62 × 10^−5^). All nominally significant SNPs at 12p11.21 locus in both GWAS (db1 and db2) were evaluated as eQTLs (Table S3). Six genes had eQTLs at this locus including DDX11 and DNM1L (Table S3). The most significant DDX11 eQTL was rs622946 (eQTL *p* = 9.23 × 10^−28^) but its genomic significance was low (*p* = 0.022), compared to rs3741869 (eQTL *p* = 8.62 × 10^−5^ and GWAS *p* = 3.2 × 10^−5^). The Z-score for rs622946 was −10.92 indicating it downregulated DDX11, whereas rs3741869 upregulated DDX11 (Z = 3.93).

### 2.3. Locus 12p11-Specific Genomic Convergence

**Figure 1:**
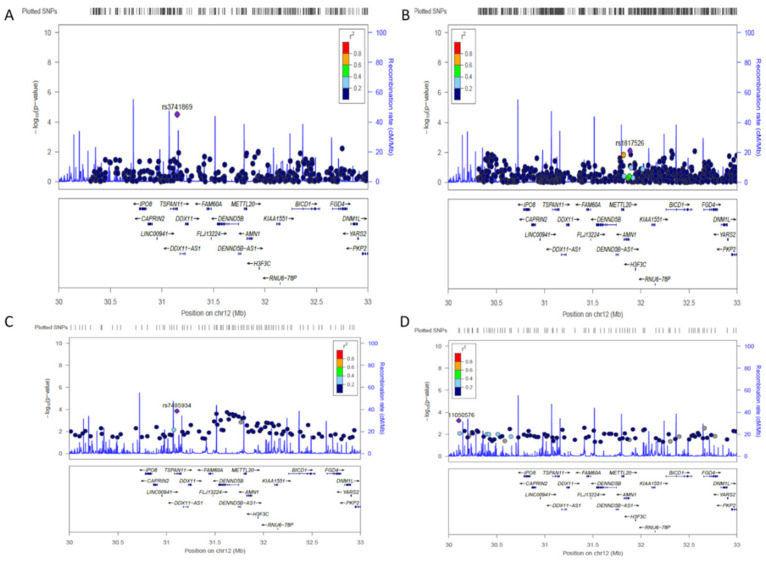
LocusZoom plot of genetic association at locus 12p11.21. (**A**) Association plot of all SNPs genotyped in GWAS db1 at the 12p11.21 locus. There are 60 genes at this locus. (**B**) LocusZoom plot of all SNPs genotyped in GWAS db2 at the 12p11.21 locus. (**C**) OASIS analysis identified the most significant SNPs in 20 kb window size which were plotted for db3 using LocusZoom. (**D**) Plot of the most significant SNPs in db4.

As 12p11 had previously been linked to SLE [7], this locus was further tested for association using OASIS in two additional GWAS (db3 and db4). Both these GWAS originally failed to identify association at this locus mainly due to statistical corrections for multiple testing. LocusZoom.js v0.12 plots showed the association signal at 12p11.21 (Figure 1A–D). In db3, there was a modest association peak near DDX11 with the most significant SNP being rs7485934 (*p* = 1.45 × 10^−4^) located at 31.1 Mb. In db4, rs11050576 located at 30.1 Mb was the most significant SNP (*p* = 5.81 × 10^−4^) though the signal at 30–33 Mb was low (ranging mostly *p* = 1 × 10^−2^ to 1 × 10^−3^) compared to db3.

Genetic association of the entire 12p11 locus in db3 and db4 showed three association peaks located at 24–26 Mb, 30–33 Mb and 33–35 Mb (Figure S1D). The first association peak containing KRAS was located upstream of the 12p11 locus and had low LD (Figure S2).

LD was also modest in the second association peak containing DDX11 and DNM1L (Figure S3). The third association peak was in a high LD, gene-sparse locus (Figure S4).

The NCBI gene database identified 150 genes at the 12p11 locus, which were tested for significant expression changes in GEO expression datasets as described above. Besides DDX11, two genes were found to be differentially expressed in at least two GEO datasets with all probes directionally aligned viz DNM1L (GSE13887: *p* = 0.046; GSE10325: *p* = 0.041) and KRAS (GSE13887: *p* = 0.03; GSE10325: *p* = 0.02). Both these genes were upregulated in SLE.

### 2.4. DDX11, DNM1L and KRAS qPCR

**Figure 2:**
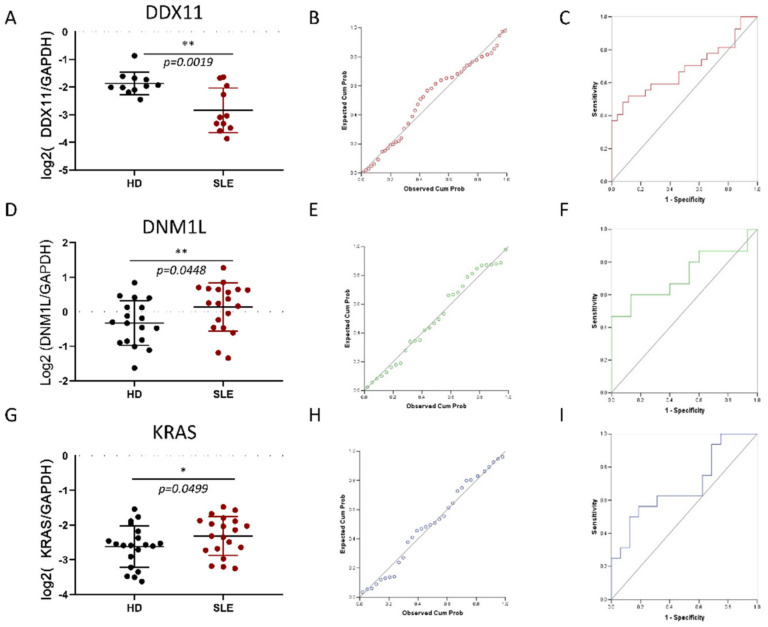
Validation of DDX11, DNM1L and KRAS expression in monocytes from SLE. (**A**–**C**) Comparison of DDX11, DNM1L and KRAS mRNA levels, respectively, between monocytes isolated from SLE patients and healthy donors (HD). Data were normalized using the expression of the control gene GAPDH, and subsequently logarithmically transformed to improve the visualization. Asterisks (* *p* < 0.05 and ** *p* < 0.01) indicate statistically significant differences between HD and SLE samples. (**D**–**F**) Receiver Operator Characteristic (ROC) curve of DDX11, DNM1L and KRAS expression. (**G**–**I**) P-P Plot of the DDX11, DNM1L and KRAS expression levels.

The clinical characteristics of SLE patients and HD are shown in Table 1. Expression of DDX11 was confirmed with qPCR in monocytes isolated from SLE patients, and HD. DDX11 was significantly repressed in SLE patients (*t*-test = 222; P2-tailed = 0.028) (Figure 2A). Data were adjusted as logarithms. A P-P plot showed slightly skewed distribution of DDX11 expression (Figure 2D). The ROC curve was significant (AUC = 0.684 ± 0.075, CI95% = 0.54–0.83, *p* = 0.022) (Figure 2G). We tested the DDX11 expression levels in SLE cases with age, SLEDAI, dsDNA levels, CRP and C3 and C4, and found no correlation. Additionally, we tested DDX11 levels in cases and controls combined, against CRP, C3 and C4, and found no correlations. Hence, DDX11 was repressed in SLE monocytes, and its expression could be differentiated between SLE and HD, functionally verifying DDX11 as a significant SLE gene.

The expression of DNM1L and KRAS was similarly investigated with qPCR in monocytes from 20 SLE cases and 20 HD controls. As shown DNM1L (*t*-test = 63; P2-tailed = 0.04) expression was significantly increased in SLE cases (ROC curve AUC = 0.72 ± 0.097, CI95% = 0.53–0.91, *p* = 0.04) (Figure 2B,E,H). KRAS (*t*-test = 80; P2-tailed = 0.07) could not be validated in this study (ROC curve AUC = 0.69 ± 0.095, CI95% = 0.5–0.88, *p* = 0.07) (Figure 2C,F,I).

### 2.5. Protein Network Analysis

**Figure 3:**
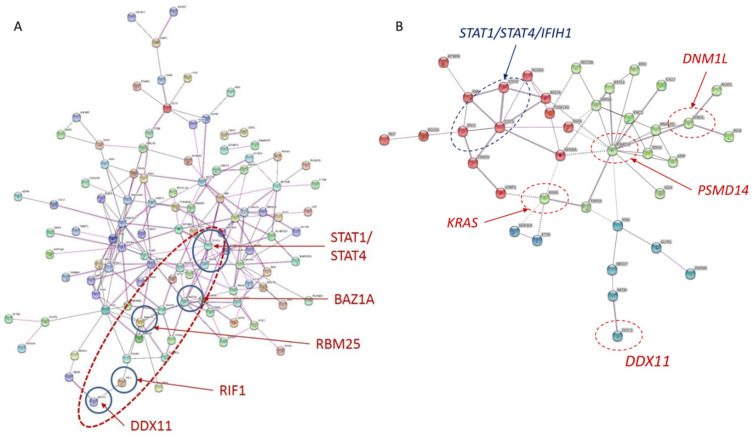
Protein network analysis. STRING protein network analysis of 187 unique genes (**A**) that have significant changes in expression in six GEO datasets and are located in the 30 SLE loci. STRING analysis of 55 genes (**B**) having significant differential expression in at least two GEO datasets and with their expression directionally aligned. DNM1L and KRAS were included in this interaction network (**B**) and cluster analysis was performed using k-means. A minimum interaction score of 0.15 was chosen for either coexpression or experimental interaction.

Protein network analysis using STRING of 187 genes (Table S2) showed a complex interaction network. DDX11 interacted indirectly with several genes found to be significant in OASIS loci and GEO datasets. DDX11 interaction with STAT1/STAT4 was mediated via RIF1, RBM25 and then BAZ1A (Figure 3A). Interestingly, the remaining 132 genes included several known SLE genes such as IRF5, BLK, TNIP1 and CD44; however, these genes were either significant in a single GEO dataset only or did not have uniform expression. STRING analysis provided further supportive evidence that DDX11 is potentially involved in SLE pathobiology.

Interaction analysis of 55 genes (Table S3), showed that DDX11 interacted directly but modestly with MED6 (interaction score 0.31) (Figure 3B). DNM1L interacted with PSMD14 (0.41), and this link connected it with KRAS via TNPO3 (0.17) (Figure 3B). Clustering using k-means into three groups showed that DDX11, DNM1L and KRAS interacted through the PSMD14 node with major SLE genes, STAT1/STAT4 and IFIH1. Interestingly, PSMD14, located in an OASIS significant locus on chromosome 2, crossed the FDR as well as Bonferroni corrections (Table S3) and is, therefore, an important candidate gene for SLE.

## 3. Discussion

This study employed a genetic and functional analysis using OASIS meta-analysis of multiple existing GWAS and gene expression datasets to identify novel SLE genes. The most important finding of this study was the identification of DDX11 and DNM1L as SLE susceptibility genes at the 12p11 locus. DDX11 was downregulated in both the GEO and the Cordoba SLE expression datasets. DDX11 downregulation in SLE was confirmed using qPCR on monocytes. The most significant SNP, rs3741869, in OASIS locus 19 containing the gene DDX11, was a cis-eQTL regulating the expression of DDX11. DNM1L was upregulated in SLE, and several SNPs associated in GWAS db1 and db2 function as eQTLs regulating its expression. Resequencing studies will identify the functional variant(s) in DDX11 and DNM1L. It may be possible that their expression is influenced by haplotypes of eQTL SNPs, rather than by a single variant. Both DDX11 and DNM1L interact with multiple known SLE genes including STAT1/STAT4 and IFIH1, all of them closely associated with SLE development, and also identified using genomic convergence of OASIS loci and expression analysis. This study provides a major step forward in the SLE genetics of 12p11 locus by extensively mapping the association signal in four independent GWAS that had originally designated this locus as negative, functionally identifying DDX11 and DNM1L as susceptibility genes whose expression is altered in SLE and identifying the potentially responsible eQTLs. Further supportive evidence of the involvement of these genes in SLE pathways was provided by protein network analysis.

The DEAD/H box helicase, DDX11, is an RNA helicase involved in genome stability. DDX11 mutations cause the Warsaw Breakage Syndrome (WABS), which shows features of genome instability similar to Fanconi anemia (FA) [8]. DDX11 functions as an FA pathway backup, and its deficiency leads to impaired strand repair [9], while its inhibition caused apoptosis in melanomas [10]. Previously, another melanoma-associated gene, Melanoma differentiation antigen 5 (MDA5), has been shown to be important in SLE [1]. Moreover, DDX11 is involved in immunoglobulin diversification [9], which makes it an even more interesting candidate gene for SLE.

DNM1L has been shown to alter mitophagy in T and B cells of SLE mouse models [11,12]. Mitophagy is autophagic removal of dysfunctional mitochondria and is important in immune regulation. Altered mitophagy led to auto-antibody production and lupus nephritis [11,12]. DNM1L expression modulates mitophagy, though heterogeneously, and needs further study [13].

Wide mapping of the 12p11 locus in four GWAS identified two further association peaks. KRAS was the only gene at 24–26 Mb (upstream of 12p11) with significantly altered expression in GEO datasets. However, this finding could not be replicated by qPCR of SLE and HD. KRAS remains an interesting SLE candidate gene because novel mutations have been identified in a few patients using next-generation sequencing [14,15,16]. The second association peak at 34 Mb is in a high LD, gene-sparse region. None of the genes located here showed significant expression change in SLE. This signal could be due to: (i) LD with DDX11 and DNM1L SLE susceptibility variant(s); (ii) resulting from population stratification or (iii) harboring a novel gene. Given the high LD in the region and its proximity to the DDX11/DNM1L locus, it is likely that the signal is due to LD with this locus.

Furthermore, this study identified several other SLE candidate genes such as PSMD14, which need additional data for verification. Hence, the replication-based genomic convergence approach with OASIS, and expression studies, can help identify novel SLE genes. This was previously not possible even with gene-based testing [2]. The identification of DDX11 and DNM1L as SLE susceptibility genes will provide further insight into SLE pathogenesis. Limitations of this study include a need for larger sample sizes to verify the expression change in DDX11 and DNM1L, and correlation with various sub-phenotypes of SLE to identify the risk phenotype. Further functional studies including proteomic, immunohistopathologic, in vitro and in vivo strategies would help to confirm the pathogenic relevance of these genes and may provide diagnostic therapeutic targets for SLE modulation. This study lays the foundation for understanding the pathogenic involvement of DDX11 and DNM1L in SLE by identifying them using a systems-biology approach, while the 12p11 locus harboring these genes was previously missed by four independent GWAS.

## 4. Materials and Methods

### 4.1. Datasets

GWAS datasets were obtained from dbGAP [Available online: https://www.ncbi.nlm.nih.gov/gap/ and https://www.ebi.ac.uk/gwas/ (accessed on 10 May 2021)]. Meta-analysis of two SLE datasets, phs0002027 (db1) and phs0001228 (db2), was conducted using OASIS (Python 2.7.9 code) [Available online: https://github.com/dr-saeed/OASIS/blob/master/OASIS.py (accessed on 10 May 2021)] [2]. The dataset phs000202 consisted of 706 SLE females and 353 controls [17], while phs000122, was comprised of 1435 SLE cases and 3583 controls genotyped for 500,000 SNPs [18]. SNIPPER [Available online: http://csg.sph.umich.edu/boehnke/snipper/ (accessed on 10 May 2021)]. was used to identify genes in OASIS loci. This list of reference genes was tested in three GEO datasets (GSE30153, GSE13887, GSE10325) for expression. Two datasets, GSE30153 (B-cell transcriptome), GSE13887 (T-cell transcriptome), had SLE cases and healthy controls only, whereas GSE10325 had three cellular fractions (T, B cells and Monocytes) resulting in three additional datasets (GSE10325: T, B, M). A fourth expression dataset, GSE50395 (monocyte transcriptome, from Cordoba was used for validation [19]. However, the expression analysis comparing SLE with controls is new. We took the approach of overlapping expression pattern in at least two cell types to maximize the possibility of discovering the most significant SLE candidate gene(s). Figure S5 details the methodology for gene discovery.

The DDX11 locus 12p11 has also been previously linked to familial SLE [7,19]. This locus was further investigated for genetic association in two additional GWAS, GCST003156 [20] (db3: 7219 SLE cases and 15,991 controls of European ancestry, with 30,737 SNPs genotyped at 12p11) and GCST005831 [21] (db4: 907 SLE patients and 1524 healthy controls from Spain, with 18,688 SNPs genotyped at 12p11). These datasets were obtained from the GWAS Catalog. At the DDX11 locus (30–33 Mb), 5914 SNPs in db3 and 7194 SNPs in db4 were genotyped. The 12p11 locus spans 26,300,001 to 35,500,000 bp and harbors 127 genes. Hence, a slightly broader search, from 25–36 Mb, identified 150 genes on NCBI Gene [Available online: https://www.ncbi.nlm.nih.gov/gene (accessed on 10 May 2021)] which are assessed for gene expression in all the GEO datasets mentioned above. This region was genotyped for 35,382 SNPs in db3 and 21,414 SNPs in db4.

### 4.2. eQTL and Statistical Analysis

Association plots of the 12p11 locus were obtained using LocusZoom.js vs0.12 [Available online: http://locuszoom.org/ (accessed on 10 May 2021)] for all SNPs in db1 and db2. However, due to the large number of SNPs in db3 and db4, the most significant SNPs in OASIS analysis using a 20 kb window were plotted with LocusZoom. Significant SNPs in loci harboring differentially expressed genes in SLE were tested using Blood eQTL Browser (expression quantitative trait loci [Available online: https://genenetwork.nl/bloodeqtlbrowser/ (accessed on 10 May 2021)] [22]. Statistical analysis was performed using SSPS V.15.0 (SPSS Inc., Chicago, IL, USA). For DDX11, DNM1L and KRAS expression, after normalization using GAPDH as internal control, statistical significance was assessed using *t*-tests. Receiver Operator Characteristic curves and P-P plots were generated using SPSS. Graphs were generated using GraphPad Prism 8.4.3.

### 4.3. Quantitative Real-Time PCR (qPCR)

Changes of DDX11 were validated in an independent cohort of 31 SLE patients and 32 healthy donors (HD) from Cordoba, Spain (Table 1) by quantitative real-time RT-PCR using a LightCycler thermal cycler system (Roche Diagnostics, Indianapolis, IN, USA), using GAPDH as housekeeping gene. Specific primers for DDX11 were previously reported [23]. These samples were collected after obtaining approval from the ethics committee of the Reina Sofia Hospital, Cordoba. All subjects provided written informed consent. Briefly, RT was performed using an NZY First-Strand cDNA Synthesis Kit Reverse Transcription Kit (Nzytech, Lisbon, Portugal), following the manufacturer’s instructions. For the qPCR, the LightCycler thermocycler system (Roche Diagnostics, Indianapolis, IN, USA) was used. The reaction was carried out with SYBR^®^ Green (Promega Biotech, Madrid, Spain) according to the manufacturer’s instructions. Expression of DDX11 was corrected by the geometric average of α-actin (ACT) and glyceraldehyde-3-phosphate dehydrogenase (GAPDH). The data were analyzed by the 2-ΔΔCt method. The expression of DNM1L and KRAS was similarly assessed in 20 SLE cases and 20 HD controls, as described for DDX11 above.

### 4.4. Protein Network Analysis

All genes with significant and consistent directional change in expression in the GEO datasets were tested for protein–protein interactions using STRING [Available online: http://string-db.org (accessed on 10 May 2021)] [24]. Gene pairs that were either coexpressed or involved in experimentally validated interactions with at least a low score (0.15) were evaluated.

## Figures and Tables

**Table 1:** Clinical and laboratory parameters for patients with systemic lupus erythematosus (SLE) and healthy donors (HD).

Parameter Mean ± SD or *n* (%)	SLE *n* = 31	Healthy Donors *n* = 32	*p*
Demographic and clinical characteristics			
Females/males, *n* (%)	25/6 (81%/19%)	25/7 (78%/22%)	ns
Age, years	41.1 ± 9.8	36.9 ± 9.0	ns
SLEDAI	1.83 ± 1.49	0	--
Lupus nephritis, *n* (%)	27/31 (87%)	0	--
Pregnancy morbidity, *n* (%)	7/25 (28%)	0	--
Hypertension, *n* (%)	7/31 (23%)	0	--
Smoker, *n* (%)	8/31 (26%)	5/32 (16%)	ns
Medications			
Corticosteroids, *n* (%)	21/31 (68%)	0	--
Antimalarials, *n* (%)	17/31 (55%)	0	--
Statins, *n* (%)	8/31 (26%)	0	--
Laboratory features			
CRP, mg/L	4.1 ± 8.1	2.9 ± 4.9	0.033
ESR, mm/h	24.41 ± 22.02	4.75 ± 1.71	0.004
C3, mg/dL	99.6 ± 32.5	105.4 ± 20.3	ns
C4, mg/dL	21.2 ± 10.7	23.5 ± 7.1	ns
anti-dsDNA Ab, *n* (%)	18/31 (58%)	0	--
aCL IgG Ab, *n* (%)	1/31 (3%)	0	--
aCL IgM Ab, *n* (%)	0	0	--
Anti-β2GP IgG Ab, *n* (%)	0	0	--
Anti-β2GP IgM Ab, *n* (%)	2/31 (6%)	0	--
LA positivity	1/31 (3%)	0	--

Values are mean ± standard deviation (SD), or *n* (%). SLEDAI = Systemic Lupus Erythematosus Disease Activity Index; HDL = high-density lipoprotein; LDL = low-density lipoprotein; ApoA = apolipoprotein A; ApoB = apolipoprotein B; CRP = C reactive protein; ESR = globular sedimentation rate; aCL Ab = anticardiolopin antibody; anti-β2GP Ab = anti-beta2-glycoprotein I antibody; LA = lupus anticoagulant.

## Data Availability

All data relevant to the study are included in the article or uploaded as Supplementary Materials. All datasets are in publicly available repositories with their accession numbers in the manuscript.

## Supplementary Materials

The Supplementary Materials are available online at https://www.mdpi.com/article/10.3390/ijms22147624/s1.

## References

[1] Saeed M. (2017). Lupus pathobiology based on genomics. Immunogenetics.

[2] Saeed M. (2017). Novel linkage disequilibrium clustering algorithm identifies new lupus genes on meta-analysis of GWAS datasets. Immunogenetics.

[3] Gateva V., Sandling J.K., Hom G., Taylor K.E., Chung S.A., Sun X., Ortmann W., Kosoy R., Ferreira R.C., Nordmark G. (2009). A large-scale replication study identifies TNIP1, PRDM1, JAZF1, UHRF1BP1 and IL10 as risk loci for systemic lupus erythematosus. Nat. Genet..

[4] Morris D.L., Sheng Y., Zhang Y., Wang Y.F., Zhu Z., Tombleson P., Chen L., Cunninghame Graham D.S., Bentham J., Roberts A.L. (2016). Genome-wide association meta-analysis in Chinese and European individuals identifies ten new loci associated with systemic lupus erythematosus. Nat. Genet..

[5] Saeed M. (2018). Genomic convergence of locus-based GWAS meta-analysis identifies AXIN1 as a novel Parkinson’s gene. Immunogenetics.

[6] Liang X., Slifer M., Martin E.R., Schnetz-Boutaud N., Bartlett J., Anderson B., Zuchner S., Gwirtsman H., Gilbert J.R., Pericak-Vance M.A. (2009). Genomic convergence to identify candidate genes for Alzheimer disease on chromosome 10. Hum. Mutat..

[7] Xing C., Sestak A.L., Kelly J.A., Nguyen K.L., Bruner G.R., Harley J.B., Gray-McGuire C. (2007). Localization and replication of the systemic lupus erythematosus linkage signal at 4p16: Interaction with 2p11, 12q24 and 19q13 in European Americans. Hum. Genet..

[8] Eppley S., Hopkin R.J., Mendelsohn B., Slavotinek A.M. (2017). Clinical Report: Warsaw Breakage Syndrome with small radii and fibulae. Am. J. Med. Genet. A.

[9] Abe T., Ooka M., Kawasumi R., Miyata K., Takata M., Hirota K., Branzei D. (2018). Warsaw breakage syndrome DDX11 helicase acts jointly with RAD17 in the repair of bulky lesions and replication through abasic sites. Proc. Natl. Acad. Sci. USA.

[10] Bhattacharya C., Wang X., Becker D. (2012). The DEAD/DEAH box helicase, DDX11, is essential for the survival of advanced melanomas. Mol. Cancer.

[11] Oaks Z., Winans T., Caza T., Fernandez D., Liu Y., Landas S.K., Banki K., Perl A. (2016). Mitochondrial Dysfunction in the Liver and Antiphospholipid Antibody Production Precede Disease Onset and Respond to Rapamycin in Lupus-Prone Mice. Arthritis Rheumatol..

[12] Caza T.N., Fernandez D.R., Talaber G., Oaks Z., Haas M., Madaio M.P., Lai Z.W., Miklossy G., Singh R.R., Chudakov D.M. (2014). HRES-1/Rab4-mediated depletion of Drp1 impairs mitochondrial homeostasis and represents a target for treatment in SLE. Ann. Rheum. Dis..

[13] Xu Y., Shen J., Ran Z. (2020). Emerging views of mitophagy in immunity and autoimmune diseases. Autophagy.

[14] Uehara T., Hosogaya N., Matsuo N., Kosaki K. (2018). Systemic lupus erythematosus in a patient with Noonan syndrome-like disorder with loose anagen hair 1: More than a chance association. Am. J. Med. Genet. A.

[15] Ragotte R.J., Dhanrajani A., Pleydell-Pearce J., Del Bel K.L., Tarailo-Graovac M., van Karnebeek C., Terry J., Senger C., McKinnon M.L., Seear M. (2017). The importance of considering monogenic causes of autoimmunity: A somatic mutation in KRAS causing pediatric Rosai-Dorfman syndrome and systemic lupus erythematosus. Clin. Immunol..

[16] Leventopoulos G., Denayer E., Makrythanasis P., Papapolychroniou C., Fryssira H. (2010). Noonan syndrome and systemic lupus erythematosus in a patient with a novel KRAS mutation. Clin. Exp. Rheumatol..

[17] Harley J.B., Alarcon-Riquelme M.E., Criswell L.A., Jacob C.O., Kimberly R.P., Moser K.L., Tsao B.P., Vyse T.J., Langefeld C.D., International Consortium for Systemic Lupus Erythematosus Genetics (SLEGEN) (2008). Genome-wide association scan in women with systemic lupus erythematosus identifies susceptibility variants in ITGAM, PXK, KIAA1542 and other loci. Nat. Genet..

[18] Hom G., Graham R.R., Modrek B., Taylor K.E., Ortmann W., Garnier S., Lee A.T., Chung S.A., Ferreira R.C., Pant P.V. (2008). Association of systemic lupus erythematosus with C8orf13-BLK and ITGAM-ITGAX. N. Engl. J. Med..

[19] Pérez-Sánchez C., Barbarroja N., Messineo S., Ruiz-Limón P., Rodríguez-Ariza A., Jiménez-Gómez Y., Khamashta M.A., Collantes-Estévez E., Cuadrado M.J., Aguirre M.A. (2015). Gene profiling reveals specific molecular pathways in the pathogenesis of atherosclerosis and cardiovascular disease in antiphospholipid syndrome, systemic lupus erythematosus and antiphospholipid syndrome with lupus. Ann. Rheum. Dis..

[20] Bentham J., Morris D.L., Graham D.S.C., Pinder C.L., Tombleson P., Behrens T.W., Martin J., Fairfax B.P., Knight J.C., Chen L. (2015). Genetic association analyses implicate aberrant regulation of innate and adaptive immunity genes in the pathogenesis of systemic lupus erythematosus. Nat. Genet..

[21] Julia A., Lopez-Longo F.J., Perez Venegas J.J., Bonas-Guarch S., Olive A., Andreu J.L., Aguirre-Zamorano M.A., Vela P., Nolla J.M., de la Fuente J.L.M. (2018). Genome-wide association study meta-analysis identifies five new loci for systemic lupus erythematosus. Arthritis Res. Ther..

[22] Westra H.J., Peters M.J., Esko T., Yaghootkar H., Schurmann C., Kettunen J., Christiansen M.W., Fairfax B.P., Schramm K., Powell J.E. (2013). Systematic identification of trans eQTLs as putative drivers of known disease associations. Nat. Genet..

[23] Hormaechea-Agulla D., Gahete M.D., Jiménez-Vacas J.M., Gómez-Gómez E., Ibáñez-Costa A., L-López F., Rivero-Cortés E., Sarmento-Cabral A., Valero-Rosa J., Carrasco-Valiente J. (2017). The oncogenic role of the In1-ghrelin splicing variant in prostate cancer aggressiveness. Mol. Cancer.

[24] Szklarczyk D., Franceschini A., Wyder S., Forslund K., Heller D., Huerta-Cepas J., Simonovic M., Roth A., Santos A., Tsafou K.P. (2015). STRING v10: Protein-protein interaction networks, integrated over the tree of life. Nucleic Acids Res..

