# Supplementary material for: Expression of DDX11 and DNM1L at the 12p11 Locus Modulates Systemic Lupus Erythematosus Susceptibility
Source: Int J Mol Sci. 2021 Jul 16;22(14):7624. doi: 10.3390/ijms22147624 (PMC8304828; doi:10.3390/ijms22147624)
Supplement: Supplementary file 1 [file ijms-22-07624-s001.zip › ijms-1235555-supplementary.pdf]

SUPPLEMENTAL MATERIAL

Original Paper

Expression of DDX11 and DNM1L at the 12p11 locus modulates Systemic Lupus Erythematosus susceptibility

Mohammad Saeed1, Alejandro Ibáñez-Costa2, Alejandra María Patiño-Trives2, Laura Muñoz-Barrera[2], Eduardo Collantes Estevez2, María Ángeles Aguirre2, Chary López-Pedrera2

1 ImmunoCure, Karachi, Pakistan
2 Rheumatology Service, IMIBIC/Reina Sofia Hospital, University of Cordoba, Cordoba, Spain

Supplementary Figures

A

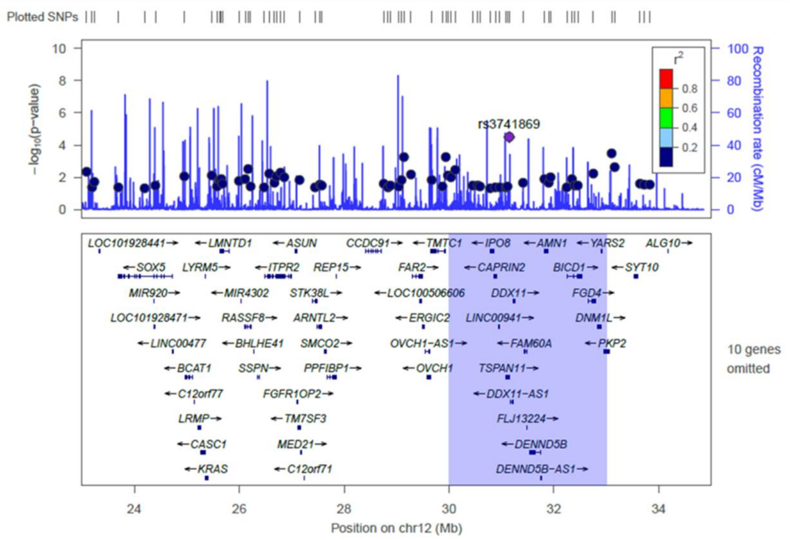

B

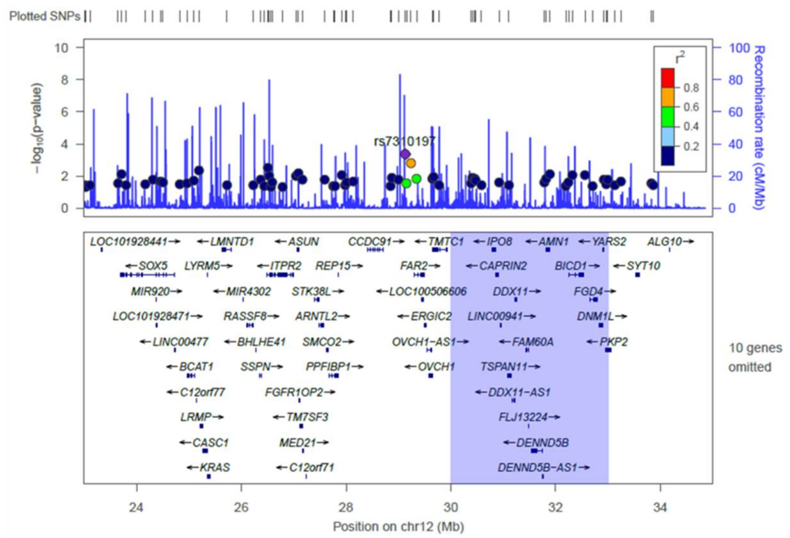

C

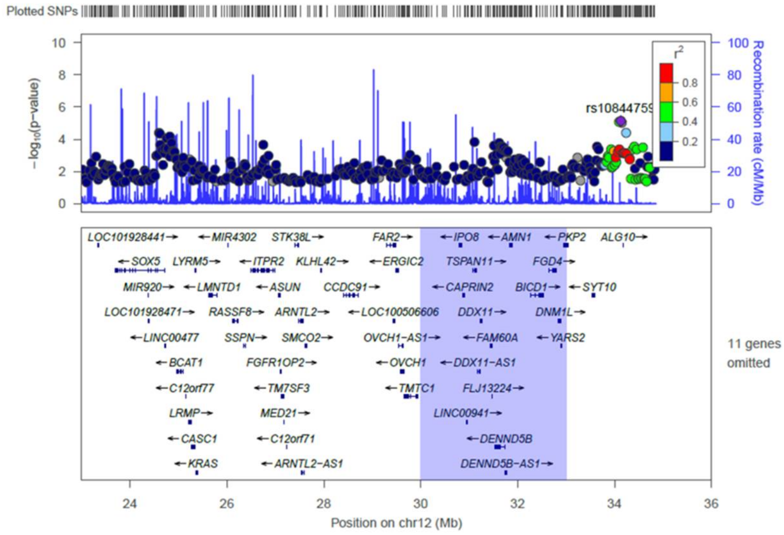

D

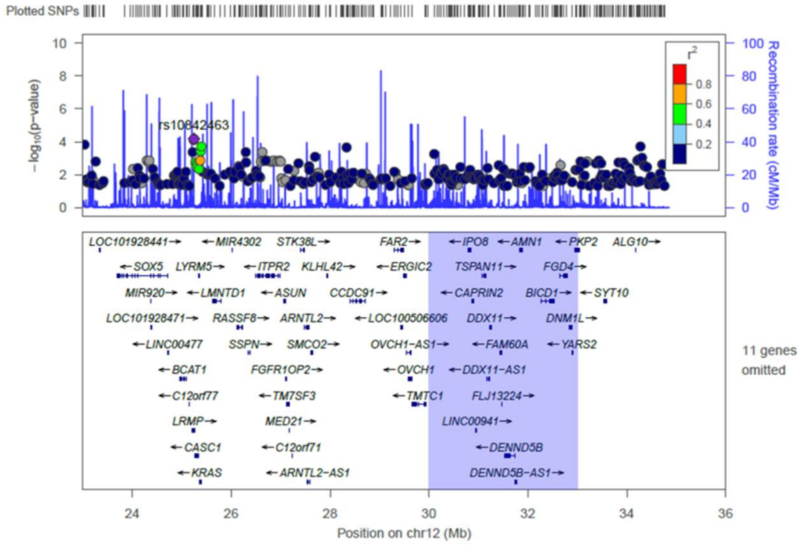

Figure S1. Association plots for the entire 12p11 locus. Most significant SNPs in OASIS analysis (20kb window size) in all four GWAS (db1 – db4) are plotted using LocusZoom. In db1, rs3741869 at 31.1Mb has the highest significance (*P*=3.19E−5). In db2, rs7310197 at 29Mb (*P*=4.35E−4) is most significant. Three association peaks are found in db3 and db4. In db3, rs11047442 (*P*=4.4E-05, 24.6Mb) and rs2230374 (*P*=2.1E-04, 26.7Mb), while in db4, rs10842463 (*P*=6.57E-05, 25.3Mb) and rs139810049 (*P*=4.1E-04, 26.3Mb) have the highest significance for the first association peak, mainly located beyond 12p11. The most significant SNPs in the second association peak are rs7485934 (*P*=1.45E-04, at 31Mb) in db3 and rs11050576 (*P*=5.8E-04, at 30Mb) in db4. The third association peak is characterized by rs10844759 (*P*=7.65E-06, at 34.1Mb) in db3 and rs10844824 (*P*=1.9E-04, at 34.3Mb) in db4.

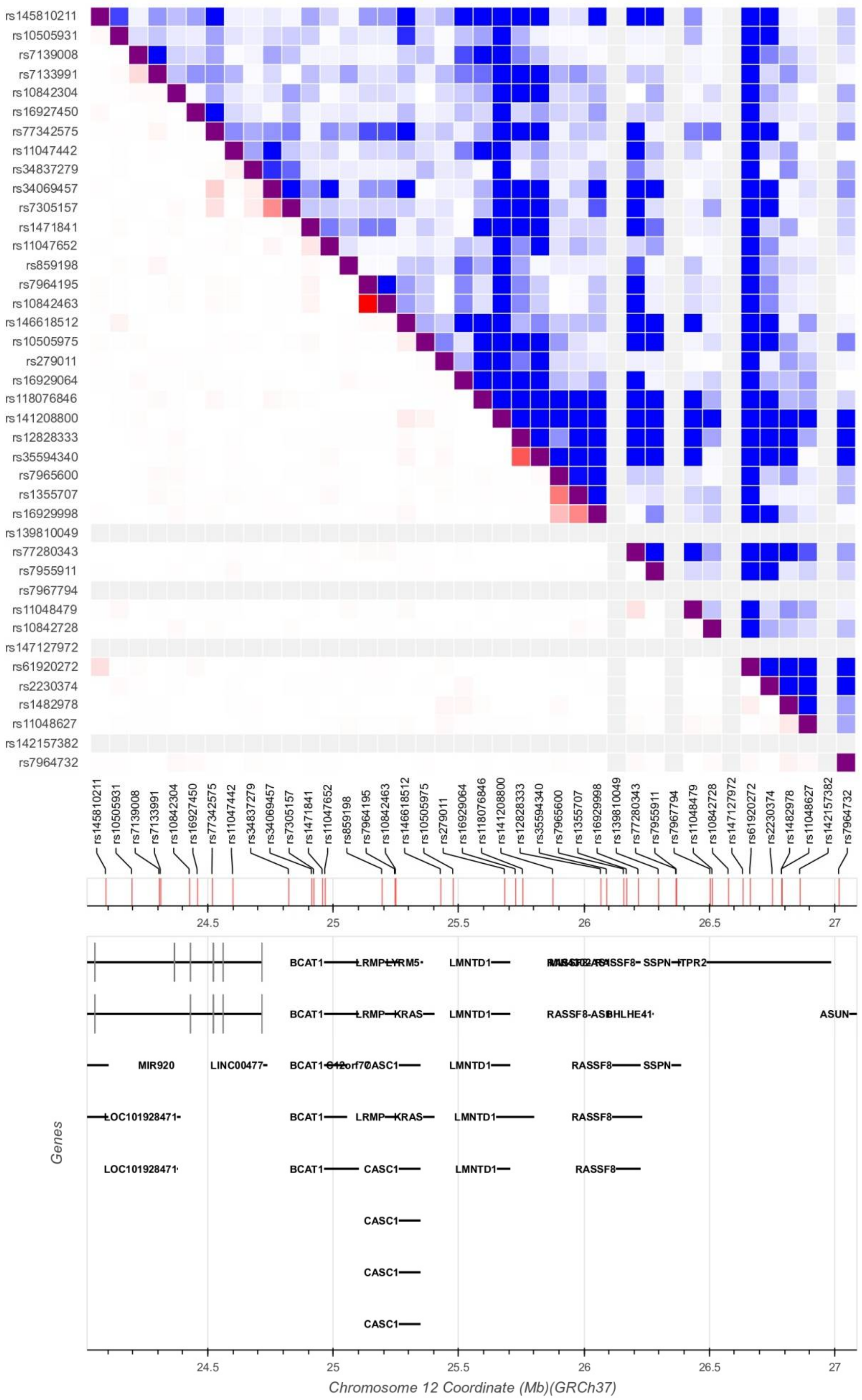
rs145810211
rs10505931
rs7139008
rs7133991
rs10842304
rs16927450
rs77342575
rs11047442
rs34837279
rs34069457
rs7305157
rs1471841
rs11047652
rs859198
rs7964195
rs10842463
rs146618512
rs10505975
rs279011
rs16929064
rs118076846
rs141208800
rs12828333
rs35594340
rs7965600
rs1355707
rs16929998
rs139810049
rs77280343
rs7955911
rs7967794
rs11048479
rs10842728
rs147127972
rs61920272
rs2230374
rs1482978
rs11048627
rs142157382
rs7964732
24.5
25
25.5
26
26.5
27
BCAT1
LRMP
LMNTD1
SSPN
ITPR2
KRAS
RASSF8-AS1
BHLHE41
ASUN
MIR920
LINC00477
C12orf77
CASC1
RASSF8
LOC101928471
Genes
Chromosome 12 Coordinate (Mb)(GRCh37)

Figure S2. Linkage disequilibrium (LD) plot for 12p 24-26Mb locus. Most significant SNPs (n=461) in all 4 GWAS (db1-db4) are identified using OASIS analysis (200kb window size) for the entire 12p11 locus. LDlink plot for the CEU population is shown for the 24-26Mb locus.

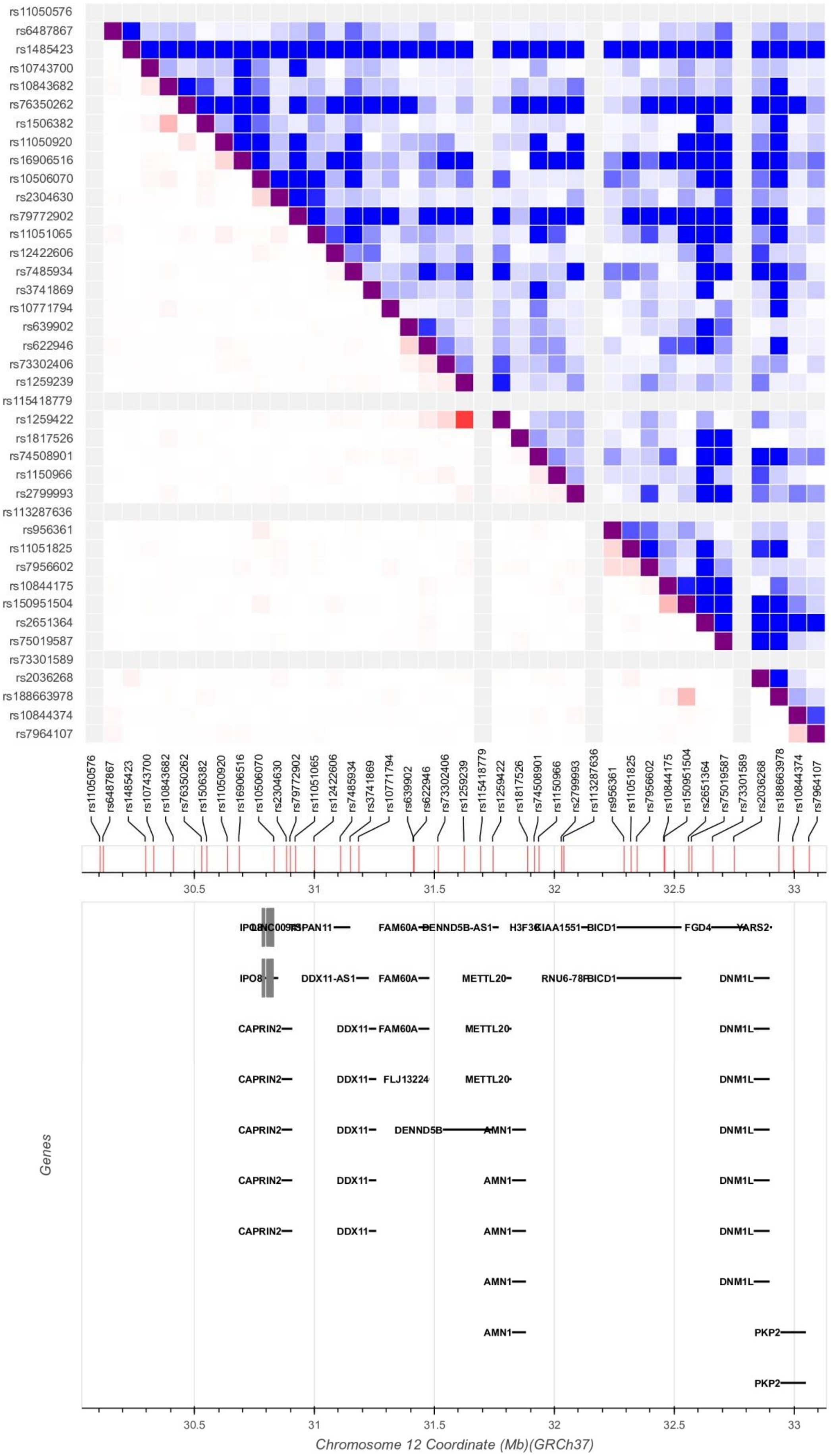

rs11050576
rs6487867
rs1485423
rs10743700
rs10843682
rs76350262
rs1506382
rs11050920
rs16906516
rs10506070
rs2304630
rs79772902
rs11051065
rs12422606
rs7485934
rs3741869
rs10771794
rs639902
rs622946
rs73302406
rs1259239
rs115418779
rs1259422
rs1817526
rs74508901
rs1150966
rs2799993
rs113287636
rs956361
rs11051825
rs7956602
rs10844175
rs150951504
rs2651364
rs75019587
rs73301589
rs2036268
rs188663978
rs10844374
rs7964107
30.5
31
31.5
32
32.5
33
TSPAN11
FAM60A
DENND5B-AS1
H3F3C
KIAA1551
BICD1
FGD4
YARS2
IPO8
DDX11-AS1
METTL20
RNU6-78P
DNM1L
CAPRIN2
DDX11
FLJ13224
DENND5B
AMN1
PKP2
Genes
Chromosome 12 Coordinate (Mb)(GRCh37)

Figure S3. Linkage disequilibrium plot for 12p 30-33Mb locus. As for Figure S3, LD plot for the most significant SNPs in 30-33Mb region in 12p11 locus is shown.

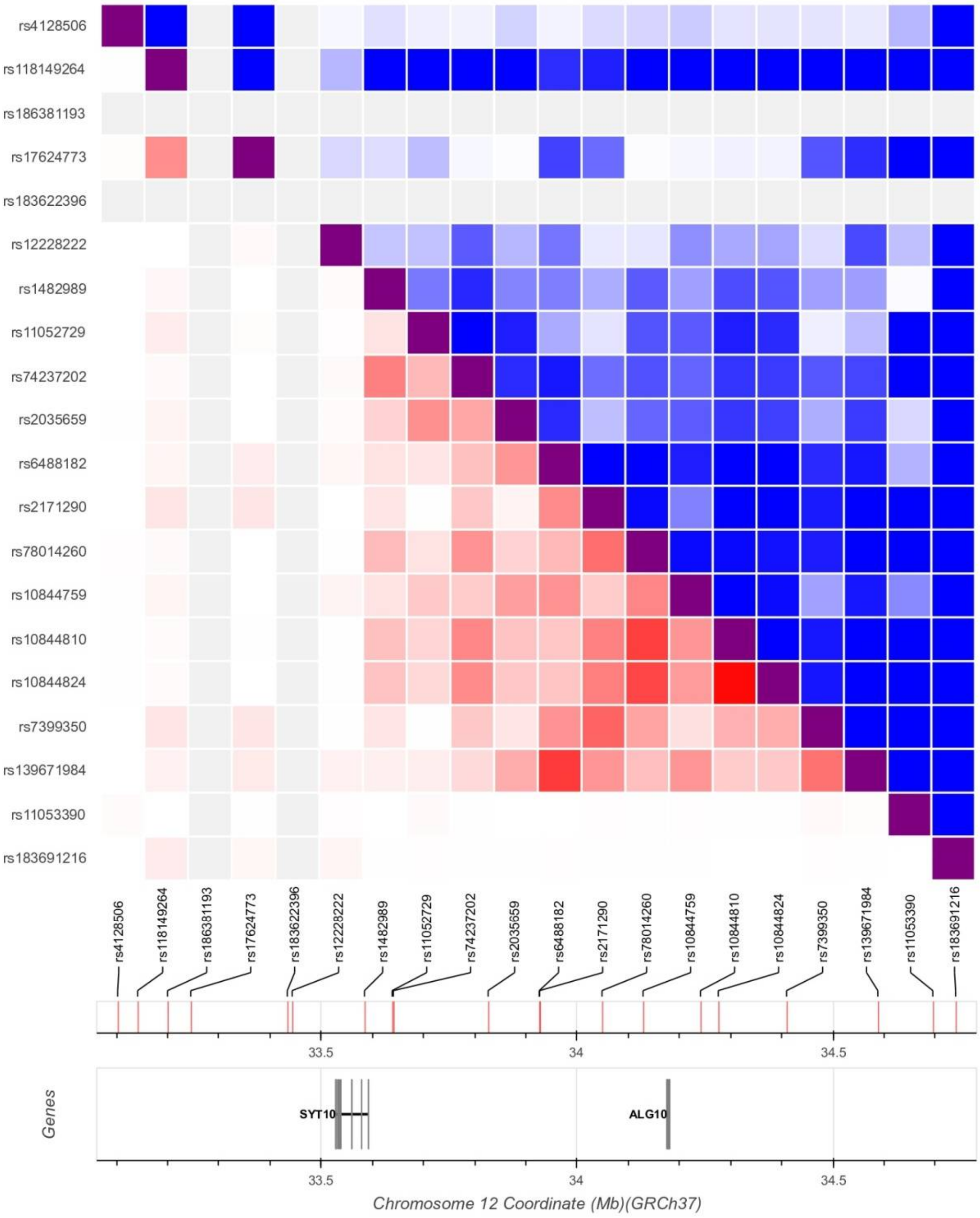

Figure S4. Linkage disequilibrium plot for 12p 33-35Mb locus. As for Figure S3, LD plot for the most significant SNPs in 33-35Mb region in 12p11 locus is shown.

Methodology for gene discovery and validation

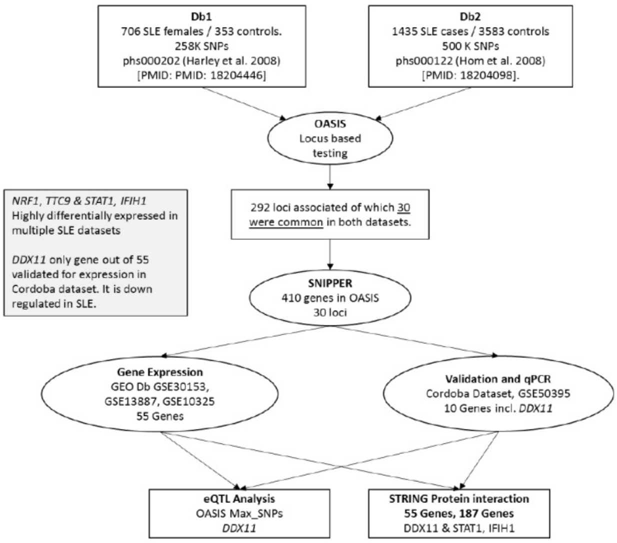

Figure S5. Methodology for gene discovery. Genomic convergence methodology employed in this study. Two GWAS (Db1 and Db2) were analyzed using OASIS locus-based testing, which identified 30 overlapping SLE loci. SNIPPER was used to identify 410 genes in these 30 loci and this reference gene list was used for gene expression analysis in 3 SLE GEO datasets and 1 dataset from Cordoba, Spain.

Supplementary Tables

Table S1. Reference list of genes in OASIS identified SLE loci. In 30 overlapping SLE loci identified by OASIS in two GWAS datasets (db1 and db2), 410 genes were found using SNIPPER.

Reference Gene List									
AAA1	C16orf89	DDX11	FKBP9	IPO8	LYPD6B	NAGPA	PSMA6	SLC36A2	TNIP1
ABLIM3	C17orf67	DDX31	FLJ13224	IRF5	MANSC1	NCOA3	PSMD14	SLC36A3	TNPO3
ABRA	C3orf67	DDX47	FLJ43663	IRGM	MAP3K9	NDST1	PYCARD	SLC39A9	TPI1P2
ABTB2	C8orf74	DEFB134	FLJ45340	ITGAD	MED27	NEB	PYDC1	SLC4A10	TRAPPC6B
ACOT1	CALU	DEFB135	FLJ45983	ITGAM	MED6	NEIL2	RAPGEF1	SLC5A2	TRIM25
ACTN1	CAMK2A	DEFB136	FLNC	ITGAX	MEST	NEMP2	RBFOX1	SLC6A7	TRIM44
ADAM20	CAPRIN2	DENND5B	FUS	ITGB6	MESTIT1	NEUROD6	RBM22	SLC8A3	TRIM72
ADAM20P1	CAT	DGKD	G3BP1	ITIH2	MFSD6	NFKBIA	RBM25	SMIM3	TRPM8
ADAM21	CBR1	DGKE	GALNT16	ITIH5	MIA2	NKAIN3	RBM43	SMO	TSC1
ADAM21P1	CBR3	DNAJB3	GATA3	ITPRID1	MIR1244-1	NMI	RBMS1	SMOC1	TSGA13
AFAP1L1	CCDC136	DOP1B	GATA4	KBTBD2	MIR1244-2	NOG	RGS6	SNORD56B	TSPAN11
AHCYL2	CCDC177	DPF3	GCA	KCNH7	MIR1244-3	NPAS3	RIF1	SNX6	TSPAN33
AHSP	CCDC69	DPP4	GCG	KCP	MIR1322	NRF1	RIOX1	SOX7	TTC9
AK8	CD44	DPY19L1P1	GEMIN2	KIAA0391	MIR143	NT5C3A	RND3	SPACA9	TTF1
AKAP1	CD74	DUSP16	GFI1B	KIN	MIR145	NTNG2	RNF126P1	SPARC	TTPA
ALG1	CDKN1B	EAPP	GGH	KLF14	MIR182	NUMB	ROGDI	SPP2	UBE2E2
ANGPT1	CDX1	EEF1AKMT1	GLRA1	KLHDC10	MIR183	OPN1SW	RP1L1	SPTSSA	UBE2H
ANKFN1	CEL	EEF2KMT	GLS	LATS2	MIR29A	OXR1	RP9	SRP54	UBN1
ANKS3	CEP41	EFNB2	GLYR1	LINC01465	MIR29B1	PAMR1	RP9P	SRSF5	UGT1A1
ANXA6	CFAP77	EGLN3	GM2A	LINC01559	MIR335	PAPLN	RPL13AP20	SSMEM1	UGT1A10
APIP	CFL2	EHF	GPR19	LOC100130705	MIR550A2	PCNX1	RPS14	STAT1	UGT1A3
APOLD1	CHAF1B	EIF3E	GPRC5A	LOC100131496	MIR598	PCTP	RSPO2	STAT4	UGT1A5
ARGLU1	CLDN14	ELF5	GPRC5D	LOC100286922	MIR613	PCYOX1L	RUNX1	STRIP2	UGT1A6
ARHGEF37	CLVS1	EMC2	GPX3	LOC100287482	MIR614	PDE1C	RUNX1-IT1	STXBP4	UGT1A7
ARMC5	CNTN5	EMP1	GRIN2B	LOC100289511	MIR802	PDE6A	SAP18	SULF2	UGT1A8
ARSI	COIL	ERH	GRPEL2	LOC145474	MIR96	PDGFRB	SCPEP1	SUSD6	UGT1A9
ASPH	COPG2	ETV6	GSG1	LOC401321	MIRLET7I	PDHX	SEC14L5	SYNJ2BP	USP15
ATOX1	COX16	EXD2	GTF3C4	LOC407835	MKLN1	PINX1	SEC23A	SYNJ2BP-COX16	USP40
ATP5F1C	COX6A2	FAM155A	GTF3C5	LOC440335	MMADHC	PLA2R1	SEPT12	SYNPO	VENTXP7
ATP6V1F	CPA1	FAM167A	HEATR4	LOC441208	MMD	PLEKHD1	SETD4	TAF3	XKR6
AVL9	CPA2	FAM167A-AS1	HEBP1	LOC643763	MON2	PLXNA4	SETX	TANK	XPO4
AVPR1A	CPA4	FAM177A1	HJURP	LOC646329	MORC3	PNN	SFMBT2	TBR1	YTHDF3
BARHL1	CPA5	FAM234B	HLCS	LOC728192	MRPL57	PODXL	SIM2	TCOF1	ZC3HC1
BAZ1A	CREBL2	FAM71F1	HLF	LOC728264	MSI2	PPARGC1B	SINHCAF	TDH	ZDHHC20
BBS9	CRYL1	FAM71F2	HMGXB3	LOH12CR2	MSL3P1	PPL	SIPA1L1	TGFB1I1	ZFYVE1
BCL2L14	CSF1R	FAP	HTR7P1	LRFN5	MTMR9	PPM1H	SKA3	TIGD6	ZMYND8
BLK	CSNK1A1	FAT2	IFIH1	LRP6	MTVR2	PPP1R17	SLC10A1	TMEM100	ZNF300

BMPER	*CTSB*	*FBXO33*	*IFT88*	*LRRC4C*	*MYO1B*	*PPP2R3C*	*SLC1A2*	*TMEM209*	*ZNF300P1*
BORCS5	*DCAF4*	*FDFT1*	*IGBP1P1*	*LSM5*	*MYOZ3*	*PRSS36*	*SLC26A2*	*TMEM74*	*ZNF385D*
C16orf58	*DCAF5*	*FHIT*	*IL17B*	*LY75*	*NAB1*	*PRSS8*	*SLC35G5*	*TMTC1*	*ZNF500*
C16orf71	*DCTN4*	*FJX1*	*IL17D*	*LYPD6*	*NABP1*	*PSEN1*	*SLC36A1*	*TNFAIP6*	*ZNF843*

Table S2. SLE candidate genes by genetic association and gene expression validation. Of the 187 candidate genes, 55 genes were differentially expressed and had consistent expression change in at least 2 GEO datasets. Genes that were statistically significant included 10 that crossed the FDR and 7 that crossed the Bonferroni correction. *STAT4* and *IFIH1* are known SLE genes and *PLA2R1* has also been previously associated. *DDX11* did not cross the FDR. In the Cordoba dataset, 10 of 518 genes with expression change were present in the reference gene list (410 genes). *DDX11* had the second highest expression change (*P*=8 x 10-3). However, only *BARHL1* (*P*=8.9 x 10-5) crossed the Bonferroni correction. Eight novel candidate genes for SLE (7 from GEO datasets and 1 from Cordoba dataset) require further data for verification.

Genetic Asociation									GEO Gene Expression						Cordoba Gene Expression		
Serial	Locus	Chr	iPos	ePos	Max_SNP db1	Max_SNP db2	Max P db1, db2	Quads db1, db2	*Gene*	Probes_Db	T_Ave	T-Min	FDR	Bonferroni	*Gene*	P-value	Fold Change Absolute
1	1	2	150459869	152099884	rs1463609	rs10490073	1.83, 3.91	C, B	***NMI***	4	3,73E-03	6,26E-04	4,16E-04	2,83E-04			
2									*TNFAIP6*	3	2,48E-02	1,04E-02	N.S	N.S			
3									*MMADHC*	2	4,55E-03	2,83E-03	N.S	N.S			
4	2	2	161182794	163311201	rs13023380	rs6755940	4.16, 3.12	B, C	***IFIH1***	8	1,41E-02	7,19E-05	8,71E-04	8,37E-04	*RBMS1*	2,39E-02	-2,88
5									***PSMD14***	4	2,13E-02	4,44E-04	5,77E-04	4,66E-04			
6									*TANK*	3	2,88E-02	2,30E-02	N.S	N.S			
7									***PLA2R1***	2	4,15E-02	4,12E-02	8,81E-03	N.S			
8	3	2	191838203	192060779	rs3771327	rs7574865	4.06, 13.05	B, A	***STAT1***	26	4,64E-03	7,75E-08	9,26E-04	9,09E-04			
9									*NABP1*	2	1,48E-02	1,02E-02	N.S	N.S			
10									*STAT4*	2	1,06E-02	7,58E-03	N.S	N.S			
11	4	2	234514762	234825092	rs4663968	rs17863787	2.61, 3.17	C, C	*DGKD*	3	3,40E-02	2,50E-02	N.S	N.S	*LOC100286922*	4,01E-02	-2,25
12	5	3	21523341	23226297	rs3860579	rs680930	3.85, 3.06	B, C	NA	NA	NA	NA	NA	NA	*ZNF385D*	3,89E-02	-4,66
13	7	5	149069958	151041931	rs2304053	rs32581	2.34, 3.24	C, C	*CSNK1A1*	16	1,77E-02	5,33E-03	N.S	N.S			
14									*SPARC*	3	4,01E-02	3,08E-02	N.S	N.S			
15									*G3BP1*	3	2,20E-02	1,28E-02	N.S	N.S			
16									*RPS14*	2	1,62E-02	7,31E-03	N.S	N.S			
17									*ATOX1*	2	4,69E-03	2,10E-03	N.S	N.S			
18	8	7	31876765	34009945	rs1362927	rs729804	4.54, 4.90	B, A	*BBS9*	4	2,62E-02	1,22E-02	N.S	N.S			
19									*AVL9*	2	3,17E-02	2,50E-02	N.S	N.S			
20	9	7	128393949	132065378	rs10488631	rs4728142	10.78, 6.14	B, B	***NRF1***	6	3,00E-02	7,98E-05	8,82E-04	8,29E-04			
21									*CALU*	5	7,07E-03	1,86E-03	N.S	N.S			
22									*TNPO3*	2	2,63E-02	8,85E-03	N.S	N.S			
23	10	8	10943275	11359637	rs13277113	rs9657519	7.12, 3.75	A, C	*MTMR9*	2	7,13E-03	5,11E-03	N.S	N.S			
24	11	8	62525895	64029901	rs4279586	rs11783343	2.95, 4.47	C, B	*GGH*	4	1,95E-02	4,18E-03	N.S	N.S			
25	12	8	107398703	109305276	rs17405978	rs1901783	3.94, 2.60	B, C	*EMC2*	3	2,36E-02	1,00E-02	N.S	N.S			
26	13	9	135083870	135453438	rs1185995	rs11243676	4.97, 5.58	B, B	*MED27*	8	2,57E-02	2,95E-03	N.S	N.S	*BARHL1*	8,85E-05	-2,66
27	14	10	7439724	7700352	rs4748940	rs11255111	7.51, 4.57	B, B	*SFMBT2*	2	2,22E-02	6,14E-03	N.S	N.S			
28	15	11	34821947	35251317	rs2785197	rs519858	3.41, 2.57	C, C	*PDHX*	3	3,26E-02	3,02E-02	N.S	N.S	*ELF5*	4,47E-02	-2,21
29									***TRIM44***	3	2,89E-02	1,02E-03	4,76E-05	N.S			
30									*APIP*	3	1,37E-02	8,86E-03	N.S	N.S			
31									*EHF*	2	2,48E-02	6,09E-03	N.S	N.S			
32	18	12	12221429	13759033	rs4764011	rs7980903	3.26, 4.39	C, B	*EMP1*	4	3,19E-02	1,33E-02	N.S	N.S	*CDKN1B*	2,76E-02	2,47
33									*ETV6*	4	1,84E-02	1,12E-02	N.S	N.S	*LOH12CR2*	4,34E-02	2,13
34	19	12	30383902	31288883	rs3741869	rs10843682	4.50, 1.89	B, C	***DDX11***	2	3,90E-02	3,60E-02	N.S	N.S	*DDX11*	8,02E-03	-2,08
35									*FLJ13224*	2	3,21E-02	2,51E-02	N.S	N.S			
36	21	13	21131210	21698119	rs7997540	rs4769124	3.98, 1.78	A, C	*XPO4*	4	2,58E-02	1,78E-02	N.S	N.S			
37	23	14	33932163	35465564	rs1201804	rs17101570	3.34, 3.06	C, C	*BAZ1A*	6	2,73E-02	1,06E-02	N.S	N.S			
38									*SRP54*	4	2,12E-02	1,62E-03	N.S	N.S			
39									*NFKBIA*	4	9,77E-03	4,58E-03	N.S	N.S			
40									*EGLN3*	2	2,60E-02	1,78E-02	N.S	N.S			
41									*SPTSSA*	2	2,53E-02	9,06E-03	N.S	N.S			
42									*SNX6*	2	2,53E-02	7,39E-03	N.S	N.S			
43	24	14	39341844	43392408	rs17113620	rs10483546	6.09, 5.02	A, B	*PNN*	3	3,04E-02	1,11E-02	N.S	N.S			
44									*SEC23A*	2	2,25E-02	1,38E-02	N.S	N.S			
45	25	14	69831036	73507392	rs2052219	rs7141009	3.09, 2.73	C, C	*MAP3K9*	3	1,99E-02	1,21E-03	N.S	N.S	*ADAM21*	2,97E-02	-3,19
46									***TTC9***	3	1,93E-02	1,20E-04	8,60E-04	7,89E-04			
47									***ERH***	3	9,23E-03	4,23E-04	5,77E-04	4,86E-04			
48									*MED6*	2	2,63E-02	1,81E-02	N.S	N.S			
49	26	16	5251130	7433109	rs10500350	rs1881335	4.12, 4.00	B, B	*ZNF500*	4	3,57E-02	1,88E-02	N.S	N.S			
50									*GLYR1*	2	2,04E-02	1,20E-03	N.S	N.S			
51	27	16	31193941	31527819	rs9888739	rs11574637	4.00, 6.27	B, B	*COX6A2*	2	2,92E-02	1,62E-02	N.S	N.S	*TRIM72*	1,79E-02	-2,30
52	28	17	53279432	55438555	rs868728	rs7503456	2.62, 4.35	C, B	*TRIM25*	3	3,94E-02	3,28E-02	N.S	N.S			
53									***AKAP1***	2	4,13E-02	3,97E-02	1,03E-02	N.S			
54									*HLF*	2	2,47E-02	1,20E-02	N.S	N.S			
55	29	20	45903384	46303730	rs6066394	rs6094661	3.82, 4.74	B, B	*NCOA3*	4	1,50E-02	8,98E-03	N.S	N.S			
56	30	21	36378570	38321199	rs6517391	rs2248159	2.37, 4.70	C, A	*RUNX1-IT1*	2	3,63E-02	3,45E-02	N.S	N.S			

Table S3. eQTL analysis of all nominally significant SNPs at the 12p11.21 locus in both GWAS. There were 71 SNPs with P<0.05 in GWAS db1 and db2 combined. Of these 26 were found in Blood eQTL Browser. All are cis-eQTLs for six genes viz *AMN1 / C12orf72, C12orf35, CAPRIN2, DDX11, DNM1L, IPO8.* Four SNPs including rs3741869, are eQTLs for *DDX11* while 6 are eQTLs for *DNM1L*. These SNPs are been highlighted. It may be possible that a haplotype of these SNPs modulates the expression of *DDX11* and *DNM1L* in SLE.

Chr	SNP	Pos	GWAS P	Gene name	eQTL P	Z-score	FDR	Cis / Trans	Probe	SNP Alleles	Minor Allele
12	rs10771720	30590795	0,04007	IPO8	4,72E-30	11,39	0	C	460411	G/A	G
12	rs2352210	30781828	0,04644	CAPRIN2	9,08E-12	6,82	0	C	6200682	C/A	A
12	rs2304630	30881808	0,04299	CAPRIN2	1,38E-12	7,09	0	C	6200682	C/T	T
12	rs11051065	30919148	0,02610063	CAPRIN2	1,18E-09	-6,08	0	C	1980202	A/G	G
12	rs2352525	30966532	0,04521	IPO8	1,20E-85	-19,61	0	C	460411	G/A	G
12	rs3741869	31148186	3,19E-05	DDX11	8,62E-05	3,93	0	C	4890750	G/A	A
12	rs35048	31162613	0,012	DDX11	8,70E-04	3,33	0,2	C	4890750	T/C	T
12	rs622946	31413324	0,02152	DDX11	9,23E-28	-10,92	0	C	4890750	G/A	A
12	rs4931478	31464662	0,03314	DDX11	8,55E-08	-5,36	0	C	4890750	A/G	G
12	rs900610	31816622	0,01521354	AMN1,C12orf72	1,24E-04	-3,84	0,1	C	6900044	A/G	A
12	rs2170768	31821260	0,01364	AMN1,C12orf72	2,25E-04	-3,69	0,1	C	6900044	C/T	C
12	rs7966525	31821823	0,01607332	AMN1,C12orf72	6,60E-05	-3,99	0	C	6900044	T/C	T
12	rs1817526	31886970	0,00824088	AMN1,C12orf72	1,15E-03	-3,25	0,3	C	6900044	T/C	T
12	rs7969019	31899732	0,02321	C12orf35	4,78E-50	14,88	0	C	7570477	C/T	T
12	rs1150965	31933212	0,01185	C12orf35	1,33E-04	-3,82	0,1	C	7510424	C/T	T
12	rs1150966	31934651	0,009936	C12orf35	1,13E-04	-3,86	0	C	7510424	T/C	C
12	rs12829317	32235034	0,04061122	C12orf35	1,28E-14	7,71	0	C	7570477	G/A	A
12	rs2652007	32244772	0,04315	C12orf35	2,08E-07	5,19	0	C	7510424	G/A	G
12	rs326633	32394103	0,03394	C12orf35	2,66E-03	3	0,4	C	7510424	T/C	C
12	rs7298165	32702739	0,04984735	DNM1L	1,06E-07	5,32	0	C	50435	T/C	C
12	rs4635166	32704907	0,0449003	DNM1L	2,95E-12	-6,98	0	C	50435	G/A	G
12	rs2036268	32748916	0,00622	-	1,92E-04	3,73	0,1	C	6350338	G/A	A
12	rs10771991	32918229	0,01830579	DNM1L	1,51E-17	8,53	0	C	50435	A/C	C
12	rs10772004	32964596	0,03161679	DNM1L	1,87E-12	7,04	0	C	50435	T/C	T
12	rs10844374	32994994	0,01716649	DNM1L	7,65E-10	6,15	0	C	50435	C/T	C
12	rs11830829	33099128	0,006166	DNM1L	7,69E-04	3,36	0,2	C	50435	G/A	G
